# Neuron-specific enolase level as a predictor of neurological outcome in near-hanging patients: A retrospective multicenter study

**DOI:** 10.1371/journal.pone.0246898

**Published:** 2021-02-10

**Authors:** Dongwook Lee, Yongil Cho, Yujin Ko, Nam Hun Heo, Hyung Goo Kang, Sangsoo Han

**Affiliations:** 1 Department of Emergency Medicine, Soonchunhyang University Cheonan Hospital, Cheonan, Republic of Korea; 2 Department of Emergency Medicine, Hanyang University Hospital, Seoul, Republic of Korea; 3 Department of Psychiatry, Soonchunhyang University Bucheon Hospital, Bucheon, Republic of Korea; 4 Department of Biostatistics, Clinical Trial Center, Soonchunhyang University Cheonan Hospital, Cheonan, Republic of Korea; 5 Department of Emergency Medicine, Soonchunhyang University Bucheon Hospital, Bucheon, Republic of Korea; University of California, Davis, UNITED STATES

## Abstract

**Objectives:**

Neuron-specific enolase (NSE) is frequently used to predict neurological outcomes in patients with hypoxic brain injury. Hanging can cause hypoxic brain damage, and survivors can suffer from neurological deficits that may impair daily activities. Here, we investigated the utility of the initial serum NSE level as a predictor of neurological outcomes in near-hanging patients with decreased consciousness.

**Methods:**

This retrospective multicenter study was conducted in patients who visited the emergency department due to near-hanging injury from October 2013 to February 2019 at three university hospitals in Korea. They were divided into two groups according to the presence of out-of-hospital cardiac arrest. The neurological outcome was determined using the Cerebral Performance Category (CPC) measured at the time of discharge. Multivariate analysis was performed to determine whether initial serum NSE is an independent predictor of neurological outcome.

**Results:**

Of the 70 patients included in the study, 44 showed a poor neurological outcome (CPC score = 3–5). Among the 52 patients with cardiac arrest, only 10 (19.2%) were discharged with good neurological outcome (CPC score = 1–2). In the whole cohort, a high serum NSE level was a significant predictor of poor neurological outcome (odds ratio [OR], 1.343; 95% confidence interval [CI], 1.003–1.800, *p* = 0.048). Among the patients with cardiac arrest, a high serum NSE level was a significant predictor of poor neurological outcome (OR, 1.138; 95% CI, 1.009–1.284, *p* = 0.036).

**Conclusions:**

In near-hanging patients, a high initial serum NSE level is an independent predictor of poor neurological outcome.

## Introduction

In the USA, the annual suicide rate due to suffocation has increased steadily from 2.7 per 100,000 in 1999–2007 to 3.7 per 100,000 in 2008–2015 [1]. In Korea, the annual suicide rate has more than doubled over the past decade, and hanging is one of the main methods of suicide, along with poisoning and falling [2]. Hanging survivors can suffer from neurological deficits that impair daily activities, and the prognosis may worsen if cardiac arrest occurs [3]. Therefore, it is important to predict the neurological outcome of near-hanging patients. Factors known to be related to the neurological outcomes of hanging include initial mental status, cardiopulmonary resuscitation (CPR) status, initial arterial blood gas analysis results, and duration of hanging [4–6].

Neuron-specific enolase (NSE) is an enzyme expressed mainly in neurons and neuroectodermal cells, which anaerobically converts glucose to metabolites suitable for oxidation [7, 8]. The serum NSE level is normally low in healthy people but increases significantly in cases of neuronal tissue damage, such as traumatic brain injury and stroke, and so is used as a biomarker for brain damage [7]. The serum NSE level also increases in cases of hypoxic brain damage; the serum concentration is proportional to the extent of brain damage [9]. As near-hanging injury causes anoxic brain injury [10], serum NSE level is expected to predict the neurological outcome of near-hanging patients. However, the prognostic value of NSE in near-hanging patients is not clear.

This study was performed to evaluate the diagnostic utility of the serum NSE level as a predictor of neurological outcomes in near-hanging patients with decreased consciousness. As the prognosis may be worse if cardiac arrest occurs, we also examined predictive factors, including the serum NSE level, according to the presence or absence of cardiac arrest.

## Materials and methods

### Study design and setting

This retrospective multicenter study was conducted in a total of 130 patients visiting the emergency department (ED) with near-hanging injury between October 2013 and February 2019 at three university hospitals in South Korea. Among all of the patients, those with International Classification of Disease (ICD) codes X70 (intentional self-harm, strangulation, and supination) and T71 (asphyxiation) were extracted. The exclusion criteria were as follows: age < 18 years, death on arrival (cardiac arrest on arrival without performance of CPR or no return of spontaneous circulation [ROSC]), alert mental status on arrival, and lack of serum NSE data. Patients were divided into two groups according to the presence of out-of-hospital cardiac arrest. The groups were further subdivided according to neurological outcome (good vs. poor). The study was approved by our institutional review board (IRB no. 2019-07-027-004) and conducted in accordance with the tenets of the Declaration of Helsinki. All data were fully anonymized before we accessed them and IRB waived the requirement for informed consent.

### Data collection

The following information was extracted from patient medical charts, including emergency medical service records, and reviewed by two emergency physicians: age, sex, vital signs (immediately after ROSC for patients with cardiac arrest) including systolic blood pressure (BP), diastolic BP, heart rate, respiratory rate, hanging time, hanging height, hanging type (complete: whole body off the ground; incomplete: some part of the body in contact with the ground), the presence of hanging marks (from the knot of the ligature at the nape of the neck), initial GCS (for patients without cardiac arrest, GCS at the time of ED arrival; for patients with cardiac arrest, GCS after ROSC), initial laboratory tests (within 24 hours of arrival) including serum NSE, pH, bicarbonate, and lactate levels, and length of stay (LOS) in hospital or the intensive care unit (ICU). For imaging studies, the diagnoses of cerebral edema and cervical spine fracture were made based on computed tomography (CT) images of the brain and cervical spine, respectively. For patients in the cardiac arrest group, CPR duration and the use of targeted temperature management (TTM) were also noted. The neurological outcome was determined based on the Cerebral Performance Category (CPC) score at the time of discharge from the hospital. A CPC score of 1–2 was classified as a good neurological outcome, and a score of 3–5 as a poor neurological outcome.

### Statistical analysis

Comparisons between the good and poor neurological outcome groups were performed using Student’s *t* test or the Mann–Whitney U test for continuous variables, after applying the Shapiro–Wilk test to determine the normality of the distribution. The chi-squared test or Fisher’s exact test was used to analyze categorical variables. Multivariable logistic regression models were used to estimate the odds ratios (ORs) for poor neurological outcomes with 95% confidence intervals (CIs). To avoid multicollinearity, the independent variable with the highest variance inflation factor (VIF) was removed (if the VIF value was < 2, we didn’t remove) [11, 12]. Variables with missing data were not included in the multivariable logistic regression model. Analyses were performed using IBM SPSS Statistics for Windows software (version 26.0; IBM Corp., Armonk, NY, USA), and *p* < 0.05 was taken to indicate statistical significance.

## Results

**Fig 1:**
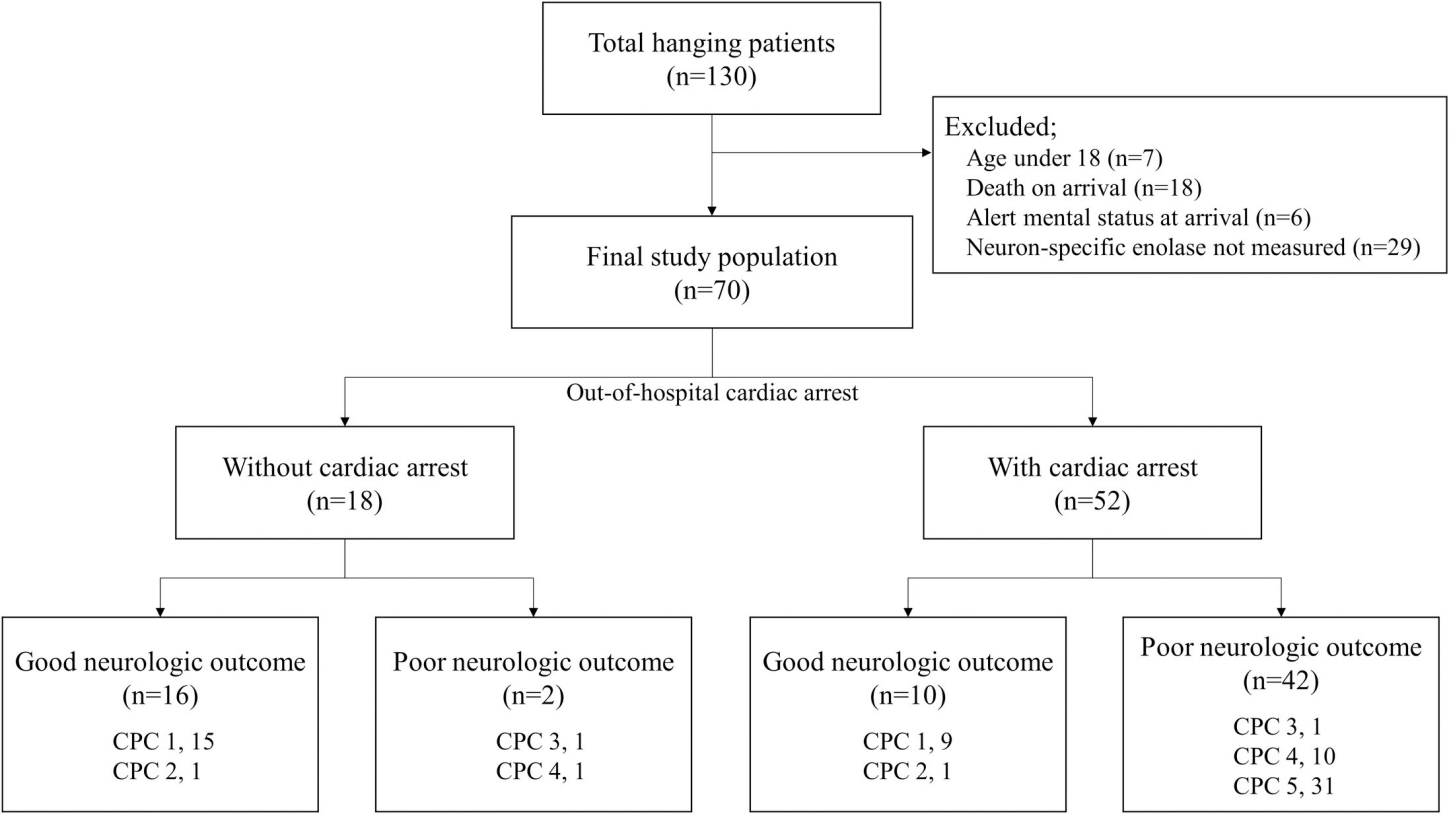
Flow diagram of the study. CPC, Cerebral Performance Category.

During the study period, a total of 130 near-hanging patients visited the ED; 60 of these patients were excluded according to the following criteria: age < 18 years (7 patients); death on arrival (18 patients); alert mental status at the time of arrival (6 patients); and serum NSE not measured (29 patients) (Fig 1). The remaining 70 patients (33 males, 47.1% and 37 females, 52.9%; mean age, 44.3 ± 14.5 years) were included in the study (S1 Table).

### Basic characteristics of near-hanging patients

Twenty-six patients had a good neurological outcome, and the remaining 44 had a poor neurological outcome. There were no significant differences in sex, age, or vital signs between the two groups. The hanging time was 15 minutes in the poor neurological outcome group, which was significantly longer than the time of 10 minutes in the good neurological outcome group (*p* = 0.01). However, there were no differences in hanging height, type, or marks between the two groups. The initial Glasgow coma scale (GCS) score was significantly lower in the poor neurological outcome group than the good neurological outcome group (3 vs. 7, respectively, *p*<0.01). With regard to initial laboratory findings, there were significant differences between the two groups in NSE level (24.1 vs. 48.65 ng/mL, respectively, *p*<0.01), pH (7.34 vs. 7.24, respectively, *p* = 0.01), bicarbonate (21.95 vs. 15.8 mmol/L, respectively, *p*<0.01), and lactate (3.5 vs. 10.25 mmol/L, respectively, *p*<0.01). The rate of cerebral edema detected on brain CT was higher in the poor neurological outcome group (29 patients, 65.9%) than the good neurological outcome group (1 patient, 3.9%). However, there was no difference in rate of cervical fracture detected on spine CT between the two groups. The rate of cardiac arrest was higher (95.5% vs. 38.5%, respectively, *p*<0.01), and LOS in the hospital (7.5 vs. 5 days, respectively, *p* = 0.03) and ICU (2 vs. 7 days, respectively, *p*<0.01) were longer, in the poor neurological outcome group than the good neurological outcome group. Table 1 compares the clinical characteristics between the good and poor neurological outcome groups (all hanging patients).

**Table 1:** Clinical characteristics of the study population.

	Neurological outcome		*p*-value
	Good	Poor	
	(*n* = 26)	(*n* = 44)	
Sex, ***n*** (%)			0.54*
Male	14 (53.9)	19 (43.2)	
Female	12 (46.1)	25 (56.8)	
Age, years	44.9 ± 16.5	43.9 ± 13.3	0.79
Vital signs			
Systolic BP, mmHg	115.5 [100.3–125.8]	110 [88.8–126.3]	0.58
Diastolic BP, mmHg	71.8 ± 17.7	68.9 ± 24.6	0.56
Heart rate, /min	94 [74.3–106.0]	100 [92.0–110.0]	0.06
Respiratory rate, /min	12 [10.5–14.8]	12 [11.5–16]	0.61
Hanging time, min	10 [5–10]	15 [10–20]	0.01
Hanging height, m	1.85 [1.2–2]	1.55 [1.5–2]	0.98
Hanging type, ***n*** (%)			0.70*
Incomplete	9 (34.6)	19 (43.2)	
Complete	14 (53.8)	21 (47.7)	
Unknown	3 (1.2)	4 (9.1)	
Hanging mark, ***n*** (%)	19 (73.1)	32 (72.7)	>0.99
GCS	7 [5–8]	3 [3–5]	<0.01
Laboratory findings			
NSE, ng/mL	24.1 [18.39–29.26]	48.65 [37.42–67.52]	<0.01
pH	7.34 ± 0.09	7.24 ± 0.18	0.01
Bicarbonate, mmol/L	21.95 [20.1–24.17]	15.8 [10.9–18.3]	<0.01
Lactate, mmol/L	3.5 [2.2–4.3]	10.25 [6.4–12.6]	<0.01
Cerebral edema on brain CT (%)	1 (3.9)	29 (65.9)	<0.01*
Fracture on cervical spine CT (%)	1 (3.9)	1 (2.3)	>0.99**
Cardiac arrest (%)	10 (38.5)	42 (95.5)	<0.01*
LOS in hospital, days	5 [3–7.5]	7.5 [4–14.8]	0.03
LOS in ICU, days	2 [1–4]	7 [4–14]	<0.01

Values are expressed as the mean ± standard deviation, median [interquartile range], or number (proportion).

*Chi-squared test

**Fisher’s exact test.

BP, blood pressure; CT, computed tomography; GCS, Glasgow coma scale; ICU, Intensive care unit; LOS, length of stay; NSE, neuron-specific enolase

### Near-hanging patients without cardiac arrest

Table 2 compares the clinical characteristics between the good and poor neurological outcome near-hanging patients without cardiac arrest. Eighteen patients did not have cardiac arrest, and only two of these patients were included in the poor neurological outcome group. The variables showing differences between the two groups were respiratory rate (12.5/min vs. 20/min, respectively, *p* = 0.03), hanging time (5 vs. 25 minutes, respectively, *p* = 0.03), and serum NSE level (21.5 vs. 49.37 ng/mL, respectively, *p* = 0.03). The hanging times of the two patients without cardiac arrest in the poor neurological outcome group were 20 and 30 minutes, respectively, and the NSE values were 53.54 and 45.2 ng/dL, respectively.

**Table 2:** Comparison between the good and poor neurological outcome near-hanging patients without cardiac arrest.

	Neurological outcome		*p*-value
	Good	Poor	
	(*n* = 16)	(*n* = 2)	
Sex, ***n*** (%)			>0.99**
Male	7 (43.8)	1 (50.0)	
Female	9 (56.2)	1 (50.0)	
Age, years	39 [33.5–61.3]	33.5 [31.3–35.8]	0.36
Vital signs			
Systolic BP, mmHg	115.5 [100.8–120.5]	100 [100–100]	0.23
Diastolic BP, mmHg	71.5 [61.5–80.3]	55 [52.5–57.5]	0.14
Heart rate, /min	85.5 [75.6–94.8]	105 [102.5–107.5]	0.16
Respiratory rate, /min	12.5 [8.0–15.0]	20 [20.0–20.0]	0.03
Hanging time, min	5 [5–10]	25 [22.5–27.5]	0.03
Hanging height, m	1.75 [1.2–2]	1.25 [1.1–1.4]	0.34
Hanging type, ***n*** (%)			0.20**
Incomplete	5 (31.3)	2 (100.0)	
Complete	8 (50.0)	0 (0.0)	
Unknown	3 (18.8)	0 (0.0)	
Hanging mark, ***n*** (%)	9 (56.3)	2 (100.0)	0.50**
GCS on arrival	8 [4.8–8]	4 [4–4]	0.13
Laboratory findings			
NSE, ng/mL	21.5 [18.55–26.72]	49.37 [47.29–51.45]	0.03
pH	7.33 [7.29–7.39]	7.41 [7.41–7.41]	0.11
Bicarbonate, mmol/L	21 [19.6–23.7]	24.1 [24.1–24.1]	0.23
Lactate, mmol/L	3.2 [1.48–3.83]	3.2 [3.2–3.2]	>0.99
Cerebral edema on brain CT (%)	1 (6.3)	0 (0.0)	>0.99**
Fracture on cervical spine CT (%)	1 (6.3)	0 (0.0)	>0.99**
LOS in hospital, days	4.5 [3–6.3]	7 [7–7]	0.23
LOS in ICU, days	2 [2–3.3]	4 [4–4]	0.17

Values are expressed as the mean ± standard deviation, median [interquartile range], or number (proportion).

*Chi-squared test

**Fisher’s exact test.

BP, blood pressure; CT, computed tomography; GCS, Glasgow coma scale; ICU, Intensive care unit; LOS, length of stay; NSE, neuron-specific enolase

### Near-hanging patients with cardiac arrest

Table 3 compares the clinical characteristics between the good and poor neurological outcome near-hanging patients with cardiac arrest. Only 10 of the 52 patients with cardiac arrest (19.2%) were discharged with a good neurological outcome. There was no difference in CPR duration between the two groups, and the GCS after ROSC was significantly higher in the good neurological outcome group than the poor neurological outcome group (5.5 vs. 3, respectively, *p* = 0.01). With regard to the laboratory test data, there were significant differences between the two groups in NSE (26.56 vs. 48.65 ng/mL, respectively, *p*<0.01), pH (7.36 vs. 7.23, respectively, *p* = 0.01), bicarbonate (22.4 vs. 15.15 mmol/L, respectively, *p*<0.01), and lactate (4.62 vs. 9.66 mmol/L, respectively, *p*<0.01) levels. The poor neurological outcome group had higher rates of cerebral edema, as detected by brain CT (69.1% vs. 0%, respectively, *p*<0.01), and TTM (88.1% vs. 30.0%, respectively, *p*<0.01); they also had a longer LOS in the ICU (7.5 vs. 2 days, respectively, *p* = 0.01).

**Table 3:** Comparison between the good and poor neurological outcome near-hanging patients with cardiac arrest.

	Neurological outcome		*p*-value
	Good	Poor	
	(*n* = 10)	(*n* = 42)	
Sex, ***n*** (%)			0.17**
Male	3 (30.0)	24 (57.1)	
Female	7 (70.0)	18 (42.9)	
Age, years	45.7 ± 17.5	44.4 ± 13.4	0.83
Vital signs			
Systolic BP, mmHg	116.4 ± 29.5	115.4 ± 41.1	0.93
Diastolic BP, mmHg	72.1 ± 19.7	69.5 ± 24.9	0.73
Heart rate, /min	95.5 [83.5–97.0]	100 [92.0–110.0]	0.13
Respiratory rate, /min	12 [12–12]	12 [10.5–15]	0.73
Hanging time, min	10 [8.8–12.5]	15 [10–20]	0.21
Hanging height, m	1.9 [1.1–2]	1.7 [1.5–2]	>0.99
Hanging type, n (%)			>0.99**
Incomplete	4 (40.0)	17 (40.5)	
Complete	6 (60.0)	21 (50.0)	
Unknown	0 (0.0)	4 (9.5)	
Hanging mark, n (%)	10 (100.0)	30 (71.4)	0.09**
CPR duration, min	15.8 ± 13.7	22.7 ± 12.5	0.17
GCS after ROSC	5.5 [5–7]	3 [3–5]	0.01
Laboratory findings			
NSE, ng/mL	26.56 [15.12–29.26]	48.65 [36.67–71.38]	<0.01
pH	7.36 ± 0.09	7.23 ± 0.18	0.01
Bicarbonate, mmol/L	22.4 [21.42–24.72]	15.15 [10.7–18.08]	<0.01
Lactate, mmol/L	4.62 ± 2.41	9.66 ± 4.16	<0.01
Cerebral edema on brain CT (%)	0 (0.0)	29 (69.1)	<0.01**
Fracture on cervical spine CT (%)	0 (0.0)	1 (2.4)	>0.99**
Underwent TTM (%)	3 (30.0)	37 (88.1)	<0.01**
LOS in hospital, days	5 [2.3–10.8]	8 [4–16.3]	0.18
LOS in ICU, days	2 [1–4]	7.5 [4–14]	0.01

Values are expressed as the mean ± standard deviation, median [interquartile range], or number (proportion).

*Chi-squared test

**Fisher’s exact test.

BP, blood pressure; CT, computed tomography; GCS, Glasgow coma scale; ICU, Intensive care unit; LOS, length of stay; NSE, neuron-specific enolase; TTM, Targeted temperature management

### Prognostic factors for poor neurological outcome

**Fig 2:**
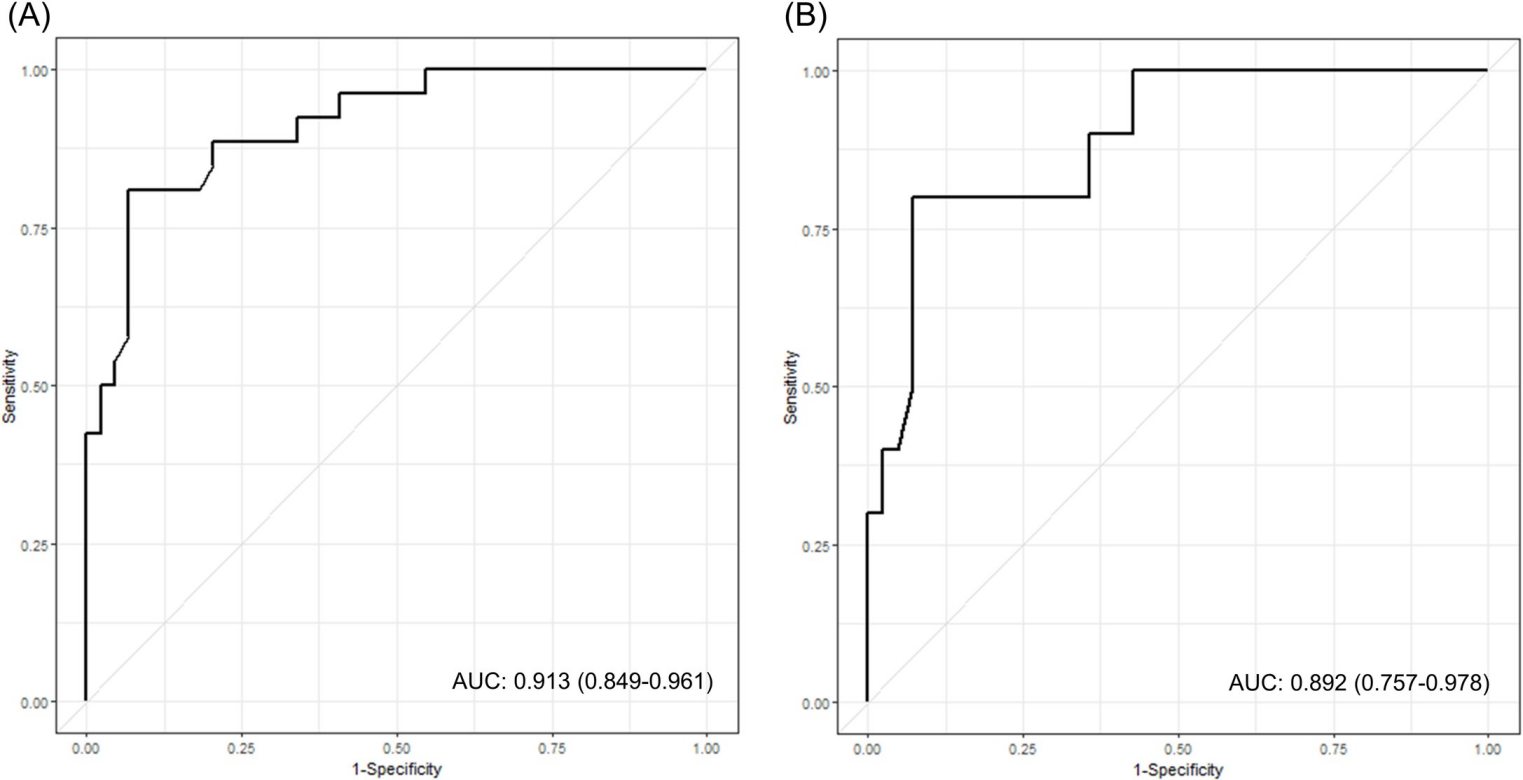
Receiver operating characteristic (ROC) curve for serum neuron-specific enolase (NSE) level and poor neurological outcome following near-hanging injury. **(A)** Predictive performance of NSE for the total patient population (AUC, 0.905; 95% CI, 0.841–0.958). **(B)** Predictive performance of NSE for patients with cardiac arrest (AUC, 0.892; 95% CI, 0.757–0.978). AUC, area under the curve; CI, confidence interval.

The presence of cardiac arrest, hanging time, initial GCS, serum NSE, bicarbonate, and lactate were analyzed by multiple logistic regression to identify predictors of poor neurological outcome in the total hanging patient population. The results indicated that a high serum NSE level (OR, 1.343; 95% CI, 1.003–1.800, *p* = 0.048) was a significant predictor of poor neurological outcome (Table 4). The area under the receiver operating characteristic (ROC) curve for serum NSE was 0.913 (95% CI, 0.849–0.961) (Fig 2A). For patients with cardiac arrest, GCS after ROSC, serum NSE, bicarbonate, and lactate were analyzed by multiple logistic regression. The results indicated that a high serum NSE level (OR, 1.138; 95% CI, 1.009–1.284, *p* = 0.036) was a significant predictor of poor neurological outcome (Table 4). The area under the ROC curve for serum NSE was 0.892 (95% CI, 0.757–0.978) (Fig 2B).

**Table 4:** Multivariable logistic regression analysis to identify variables predicting a poor neurological outcome.

	Total		With cardiac arrest	
	OR (95% CI)	*p*-value	OR (95% CI)	*p*-value
Presence of cardiac arrest	26.709 (0.058–12311.58)	0.294		
Hanging time, min	1.180 (0.905–1.537)	0.221		
GCS	1.376 (0.460–4.112)	0.568	0.666 (0.320–1.385)	0.277
NSE, ng/mL	1.343 (1.003–1.800)	0.048	1.138 (1.009–1.284)	0.036
Bicarbonate, mmol/L	0.967 (0.842–1.112)	0.640	0.975 (0.913–1.041)	0.448
Lactate, mmol/L	1.473 (0.820–2.650)	0.195	1.327 (0.999–1.763)	0.051

CI, confidence interval; GCS, Glasgow coma scale; NSE, neuron-specific enolase; OR, odds ratio

## Discussion

This study was performed to identify early predictive factors of the neurological outcome in near-hanging patients with decreased consciousness. As cardiac arrest can result in a poorer prognosis, we divided the groups according to the presence or absence of out-of-hospital cardiac arrest. A high serum NSE level was shown to be a significant early predictor of poor neurological outcome in the total study population, and in near-hanging patients with cardiac arrest. To our knowledge, this is the first study to analyze the relationship between serum NSE and neurological outcome in near-hanging patients.

NSE may be released under conditions of neuronal cell injury, and hanging induces brain injury by several mechanisms. First, respiratory asphyxia occurs via complete or subacute obstruction of air flow due to strangulation, resulting in brain injury [13]. Second, hypoxic brain damage can occur when the arterial blood supply to the brain is rapidly blocked by ligature of both bilateral common carotid and vertebral arteries [14]. Third, in cases with cardiac arrest, cerebral injury could occur due to reperfusion in the hours and days after ROSC [15]. Thus, brain injury occurs in cases of near-hanging injury, thus increasing the serum NSE concentration. The serum NSE level was high in the near-hanging patients in the present study.

In this study, we checked Cerebral Performance Category (CPC) score at discharge to evaluate neurological outcome. The CPC score is one of the commonly used methods to examine the primary neurological outcome in cardiac arrest studies and the patients’ mental status, daily function, and overall cerebral function including cognitive and executive function are examined [16]. The CPC score consists of a five-point: CPC 1, good recovery; 2, moderate disability; 3, severe disability; 4, vegetative state; and 5, death due to a comatose state or brain death [17, 18].

A previous study on near-hanging injury indicated that lower GCS on arrival at the hospital and longer hanging time were associated with a poor neurological outcome [6]. However, as hanging is mostly associated with attempted suicide, it may be accompanied by intake of alcohol or sedative drugs, resulting in impaired consciousness, thus making the GCS score unreliable [19]. In addition, the hanging time may be inaccurate because it is based on witness statements. In the present study, GCS score and hanging time were not related to neurological outcome in multiple logistic regression model. Another study reported that lower arterial pH and bicarbonate levels were associated with poor neurological outcome in near-hanging patients [5]. However, in the present study, only serum NSE showed to be associated with neurological outcome in the multiple logistic regression model.

The main strength of this study was that the near-hanging patients were grouped according to the presence or absence of out-of-hospital cardiac arrest, thus yielding more practical clinical information. In previous studies on near-hanging injury without cardiac arrest, most patients were discharged with a good neurological outcome (CPC score = 1) with conservative care alone [2, 20]. Similar results were obtained in the present study. Only two (11.1%) of the patients without cardiac arrest were discharged with a poor neurological outcome, and the serum NSE level was high in both of these patients. Therefore, even in the absence of cardiac arrest in cases of near-hanging injury, physicians should prepare for a poor neurological outcome when the serum NSE level is high. On the other hand, in one study of near-hanging patients with cardiac arrest, 94.3% were discharged with a poor neurological outcome (CPC score = 3–5) [3]. In the present study, 42 of 52 (80.8%) patients with cardiac arrest were discharged with a poor neurological outcome. It is not clear whether TTM can improve the neurological prognosis in near-hanging patients, but TTM is known to be effective in reducing neurological damage in cases of cardiac arrest [3, 21]. Therefore, TTM may also be useful in near-hanging patients with cardiac arrest, and should be considered in patients expected to have a poor neurological prognosis. In addition, high serum NSE was shown to have significant utility for predicting a poor neurological outcome in the present study. Another strength of this study is that we employed a serum NSE level to predict the prognosis and as a serum index, it is more convenient, easy to be sampled, and low-invasive than other imaging tools like brain MRI and electroencephalography. Also, this is a multicenter study conducted at the three university hospitals and, as far as we know, it is the first study on relationship between the serum NSE and neurological outcome in near-hanging patients.

This study had several limitations. First, it used a retrospective chart review design. Therefore, some variables related to the hanging event may have been omitted from the analyses, and the charts may have contained inaccurate information because they were based on witness statements. Second, neurological outcome was determined by the CPC score at discharge, but this may not reflect the long-term neurological outcome. Third, the initial serum NSE level was measured within 24 hours of arrival at the hospital. The serum NSE level, measured to predict neurological prognosis in cardiac arrest patients, was reportedly highest at 48 hours after cardiac arrest [22, 23]. Therefore, in our near-hanging patients, a stronger causal relationship between NSE and poor neurological outcome may have been found if multiple serial measurements had been conducted over the 48-hour post-injury period. Fourth, this study had a small sample size. Fifth, other biochemical markers specific for brain damage were not checked. S100b, like NSE, could reflect brain damage in post-cardiac arrest and stroke [24, 25]. However, previous study reported serum S100b level is not enough to predict poor neurological outcome in hanging patient [26]. Seventh, although this was a multicenter study, only two patients without cardiac arrest had a poor neurological outcome. Therefore, we were unable to perform a regression analysis of patients without cardiac arrest. Further well-designed, large-scale studies are needed to overcome these limitations.

## Conclusions

Serum NSE is known as one of the enzymes related to brain damage, and it is easy to be sampled, low-invasive and convenient. In this retrospective multicenter study, serum NSE level was associated with neurological outcome in cases of near-hanging injury. A high serum NSE level was shown to be a useful predictor of poor neurological outcome in near-hanging injury patients. Identifying prognostic factors for poor neurological outcome may assist in planning early interventions, such as TTM, in patients expected to have a poor prognosis.

## Supporting information

S1 Table Comparison between the female and male group in near-hanging patients. (DOCX)
