# Supplementary material for: Neuron-specific enolase level as a predictor of neurological outcome in near-hanging patients: A retrospective multicenter study
Source: PLoS One. 2021 Feb 10;16(2):e0246898. doi: 10.1371/journal.pone.0246898 (PMC7875384; doi:10.1371/journal.pone.0246898)
Supplement: S1 Table — (DOCX) [file pone.0246898.s001.docx]

**S1 Table. Comparison between the female and male group in near-hanging patients**

|  | **Female** | **Male** | ***p*-value** |
| --- | --- | --- | --- |
|  | **(*n*=37)** | **(*n*=33)** |  |
| Age, years | 46 [34–58] | 39 [32–51] | 0.57 |
| Vital signs |  |  |  |
| Systolic BP, mmHg | 118.73 ± 35.77 | 110.76 ± 33.79 | 0.34 |
| Diastolic BP, mmHg | 72.68 ± 21.32 | 66.91 ± 23.01 | 0.28 |
| Heart rate, /min | 100 [92–112] | 94 [80–107] | 0.08 |
| Respiratory rate, /min | 12 [12–15] | 12 [8–14] | 0.32 |
| Hanging time, min | 10 [7–15] | 15 [8–20] | 0.50 |
| Hanging height, m | 1.5 [1.5–2] | 1.85 [1.2–2] | 0.70 |
| Hanging type, ***n*** (%) |  |  | 0.35 |
| Incomplete | 17 (45.9) | 11 (33.3) |  |
| Complete | 16 (43.2) | 19 (57.6) |  |
| Unknown | 4 (10.9) | 3 (9.1) |  |
| Hanging mark, ***n*** (%) | 27 (73.0) | 24 (72.7) | >0.99 |
| GCS | 4 [3–5] | 5 [3–6] | 0.49 |
| Laboratory findings |  |  |  |
| NSE, ng/mL | 38.59 [28.55–54.22] | 37.8 [24.9–53.54] | 0.53 |
| pH | 7.26 ± 0.17 | 7.3 ± 0.14 | 0.25 |
| Bicarbonate, mmol/L | 18 [12.5–23.6] | 19.1 [16.2–21.3] | 0.89 |
| Lactate, mmol/L | 8.8 [3.6–11.33] | 5.3 [3.1–9.8] | 0.14 |
| Cerebral edema on brain CT (%) | 15 (40.5) | 15 (45.5) | 0.86 |
| Fracture on cervical spine CT (%) | 2 (5.4) | 0 (0) | 0.49 |
| Cardiac arrest (%) | 27 (73.0) | 25 (75.8) | >0.99 |
| LOS in hospital, days | 5 [3–11] | 7 [4–13] | 0.20 |
| LOS in ICU, days | 4 [3–8] | 4 [3–13] | 0.68 |
| Neurologic outcomes, ***n*** (%) |  |  | 0.54 |
| Poor | 25 (67.6) | 19 (57.6) |  |
| Good | 12 (32.4) | 14 (42.4) |  |

Values are expressed as the mean ± standard deviation, median [interquartile range], or number (proportion). BP, blood pressure; CT, computed tomography; GCS, Glasgow coma scale; ICU, Intensive care unit; LOS, length of stay; NSE, neuron-specific enolase
